# Public health round-up

**DOI:** 10.2471/BLT.15.011115

**Published:** 2015-11-01

**Kunduz hospital bombing**

Health workers in one of the remaining parts of the Médecins Sans Frontières (MSF) Trauma Centre in Kunduz, Afghanistan after it was bombed on 3 October. At least 22 people died including 12 MSF staff and 10 patients. http://www.msf.ch/

**Figure:**
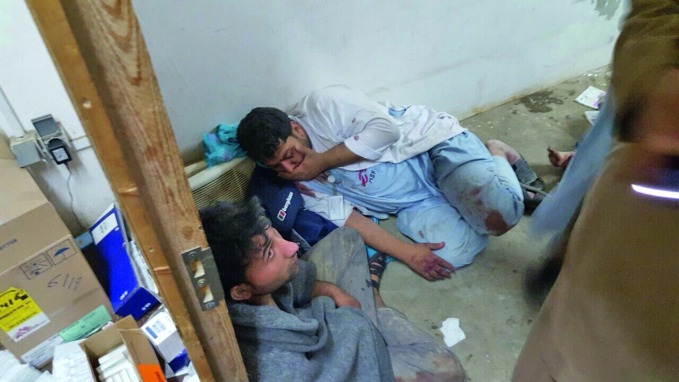
©MSF

## Call to action on climate change 

The World Health Organization (WHO) invited health professionals and others to sign up to a call to action on climate change and health that will be presented at the United Nations Climate Change Conference in Paris. 

The aim is to send a clear message to the Paris summit that health should be at the heart of the climate negotiations.

 Anyone can sign up and join the call to action which makes three key demands.

 One, strong and effective action to limit climate change, and avoid unacceptable risks to global health. Two, scaling up of financing for adaptation to climate change: including public health measures to reduce the risks from extreme weather events, infectious disease, diminishing water supplies and food insecurity.

Three, actions that both reduce climate change and improve health, including reducing the number of deaths from cancer, respiratory and cardiovascular diseases that are caused by air pollution (currently over 7 million a year).

http://who.int/globalchange/global-campaign/call-for-action/en/

## Country profiles for Paris summit 

The first 10 in a series of climate and health profiles of WHO Member States will be released this month. 

The profiles, produced in collaboration with the United Nations Framework Convention on Climate Change, can be used by policy-makers to prevent some of the adverse effects of climate change on the health of their populations. 

Additional profiles will be released ahead of the United Nations Climate Change Conference in Paris, France, from 30 November until 11 December. 

The profiles provide extensive scientific data that can be used as a baseline for tracking progress on countries’ adaptation and mitigation policies.

Adaption policies involve anticipating the adverse health effects of climate change and preventing these in the future. For example, establishing early warning systems for outbreaks of climate-sensitive infectious diseases. 

Mitigation policies aim to reduce the negative health effects of climate change, for example, levying road traffic congestion charges to reduce air pollution. 

In addition, some mitigation policies seek further health benefits. For example, encouraging people to cycle rather than drive their cars to work reduces carbon emissions, makes the air cleaner and people healthier.

The profiles are accompanied by an overview report on the consequences of addressing the adverse health effects of global warming, and of failing to do so.

http://www.who.int/globalchange/resources/country-profile/en/

## Longer not always healthier life 

Life expectancy is rising steadily across WHO’s 53-country European Region but levels of alcohol consumption, tobacco use as well as overweight and obesity – among major risk factors for premature mortality – remain alarmingly high, according to a new report.

In the *European health report 2015*, countries acknowledge the urgent need to address these problems and governments have made progress with implementing policies to tackle the risk factors, leading to declining trends in tobacco use and alcohol consumption in Europe. 

There have been improvements in tackling the main risks for premature death – cancer, diabetes, cardiovascular disease and chronic respiratory disease. 

But the decline in tobacco use for most countries is not sufficient to meet the 30% reduction target of the global noncommunicable diseases monitoring framework by 2025. 

“The European Region has the highest levels of alcohol consumption and tobacco use in the world, and ranks only slightly behind the Region of the Americas – the WHO region with the highest prevalence – in rates of overweight and obesity,” the report said. 

The prevalence of overweight – a body mass index of 25 and above – ranges, in European countries, from 45% to 67%. 

Few countries report regularly to WHO on risk factors, so the *European health report* *2015* uses WHO estimates for tobacco use and overweight and obesity. In addition, only a limited number of countries have reported mortality data to WHO in recent years.

http://www.euro.who.int/en/data-and-evidence/european-health-report/european-health-report-2015/ehr2015

**Cover photo**

A boy drags his possessions through the flooded streets of the capital of the Philippines, Manila in 2009. Floods – one form of extreme weather event associated with global warming – contaminate freshwater supplies, increase the risk of water-borne diseases and create breeding grounds for disease-carrying insects such as mosquitoes. Flooding can also lead to drowning and injuries.

**Figure:**
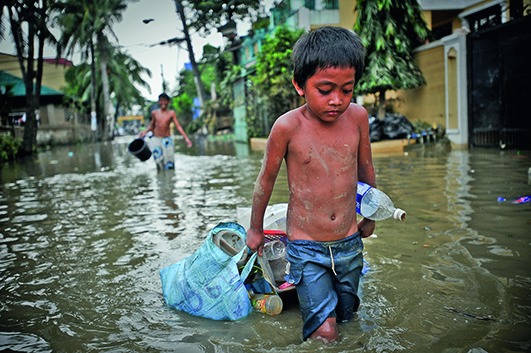
Asian Development Bank/Eric Sales

## New approach to adolescent health 

WHO and the Joint United Nations Programme on HIV/AIDS (UNAIDS) released new global standards that health-care decision-makers, managers and health-care providers can use to improve the quality of health care for adolescents.

Health services in many countries fall short of addressing the health needs of adolescents (10–19-year-olds) – a large and growing group in many developing countries – who do not always receive the care they need.

The WHO/UNAIDS *Global Standards for quality health care services for adolescents* describes the simple steps countries can take to address the needs of adolescents in many areas, including mental health, injuries, sexual and reproductive health, and substance use. 

In the past, initiatives to improve the quality of adolescent health services have tended to focus on sexual and reproductive health. 

The standards, released last month, propose a new approach and call for an integrated package of information, counselling, diagnostic, treatment and care services.

Services should be more adolescent friendly by providing free or low-cost medical consultations that are confidential. 

Health services can be made more accessible to adolescents, by providing medical consultations without having to make an appointment. Current policies on age of consent to reduce age-related barriers to services may need to be revised.

“Training health workers is critical for the successful adoption of the new approach in countries,” said Dr Valentina Baltag, from the WHO department of Maternal, Newborn, Child and Adolescent Health. 

“Adolescents are not simply ‘older children’ or ‘younger adults’, and responding to adolescents’ uniqueness requires providers to develop technical and attitudinal competencies in adolescent health,” she said. 

The new standards are based on research from many countries, consultations with health providers and feedback from more than 1000 adolescents worldwide. 

http://www.who.int/maternal_child_adolescent/documents/global-standards-adolescent-care/en/

## Treat-all recommendation for HIV 

Anyone infected with HIV should begin antiretroviral treatment (ART) as soon after diagnosis as possible while anyone at substantial risk of HIV infection should be offered preventive ART, according to a new WHO guideline. 

The new “treat-all” approach removes all limitations on eligibility for (ART) among people living with HIV. The new recommendation is based on recent scientific findings that early use of antiretroviral therapy keeps people with HIV infection alive and healthier, while reducing the risk of infecting partners with HIV.

Following further evidence of the effectiveness and acceptability of using a combination of antiretroviral drugs to prevent HIV acquisition (termed pre-exposure prophylaxis, or PrEP), WHO also recommends extending the offer of PrEP to HIV negative persons at substantial risk of acquiring HIV. PrEP should be seen as an additional prevention choice within a comprehensive package of prevention services, including HIV testing, counselling and support, and access to condoms and safe injection equipment.

Both recommendations build on previous *2013 WHO Guidelines on the use of antiretroviral drugs for treating and preventing HIV infection*, and are published ahead of a full guidelines update planned for early 2016.

Expanding ART to all people living with HIV and expanding prevention choices can help avert 21 million AIDS-related deaths and 28 million new infections by 2030, WHO said. 

http://www.who.int/hiv/pub/guidelines/earlyrelease-arv/en/

## Road safety conference in Brazil 

More than one thousand delegates from WHO Member States, United Nations agencies, civil society and business are due to gather in Brasilia, Brazil, this month to review progress and decide on the next steps to achieve global road safety goals. 

Delegates will discuss the strategies and actions needed to achieve the target 50% reduction in deaths and injuries from road traffic crashes envisaged by the Sustainable Development Goals 2015–2030 and review progress made in achieving the goals of the Global Plan for the Decade of Action from 2011 to 2020.

Hosted by the Government of Brazil and supported by WHO, the Second Global High-Level Conference on Road Safety is set to take place from 18 to 19 November. 

http://www.roadsafetybrazil.com.br/en

**Looking ahead**

14 November – World Diabetes Day.

30 November–11 December. United Nations Climate Change Conference in Paris, France.

1 December – World AIDS Day.

3 December – The International Day of Persons with Disabilities is dedicated this year to the theme "access and empowerment for people of all abilities".

